# Supplementary material for: Impact of simultaneous exposure to arboviruses on infection and transmission by Aedes aegypti mosquitoes
Source: Nat Commun. 2017 May 19;8:15412. doi: 10.1038/ncomms15412 (PMC5454532; doi:10.1038/ncomms15412)
Supplement: Supplementary Information — Supplementary Figures, Supplementary Tables and Supplementary References [file ncomms15412-s1.pdf]

**Supplementary Table 1. Infectious virus validations.**

| Experiment | Time | Condition | Sample # | PCR positive* | Infectious virus* |
|---|---|---|---|---|---|
| **Coinfection 1** | 7dpi | CHIKV/ZIKV | 62 | C, Z | C |
| | | CHIKV/ZIKV | 42 | C | C |
| | | CHIKV/ZIKV | 48 | C | C |
| | | CHIKV/ZIKV | 49 | Z | Z |
| | | CHIKV/ZIKV | 44 | neg | neg |
| | 14dpi | CHIKV/ZIKV | 100 | C, Z | C, Z |
| | | CHIKV/ZIKV | 102 | C, Z | neg |
| | | CHIKV/ZIKV | 82 | C | C |
| | | CHIKV/ZIKV | 103 | C | C |
| | | CHIKV/ZIKV | 89 | Z | Z |
| | | CHIKV/ZIKV | 96 | neg | neg |
| **Coinfection 2** | 14dpi | CHIKV/ZIKV | 1 | C, Z | neg |
| | | CHIKV/ZIKV | 19 | C, Z | C, Z |
| | | CHIKV/ZIKV | 21 | C, Z | C, Z |
| | | CHIKV/ZIKV | 7 | C | C |
| | | CHIKV/ZIKV | 11 | C | C |
| | | CHIKV/ZIKV | 38 | Z | neg |
| | | CHIKV/ZIKV | 14 | neg | neg |
| **Coinfection 3** | 7dpi | CHIKV/DENV | 37 | C, D | C |
| | | CHIKV/DENV | 38 | C, D | C,D |
| | | CHIKV/DENV | 1 | C | C |
| | | CHIKV/DENV | 2 | C | C |
| | | CHIKV/DENV | 30 | D | neg |
| | | CHIKV/DENV | 10 | neg | neg |
| | 14dpi | CHIKV/DENV | 41 | C, D | C, D |
| | | CHIKV/DENV | 48 | C | C |
| | | CHIKV/DENV | 80 | C | C |
| | | CHIKV/DENV | 53 | D | neg |
| | | CHIKV/DENV | 43 | neg | neg |
| **Coinfection 4** | 3dpi | CHIKV/ZIKV | 18 | C, Z | C,Z |
| | | CHIKV/ZIKV | 2 | C | C |
| | | CHIKV/ZIKV | 5 | C | C |
| | | CHIKV/ZIKV | 19 | C | C |
| | | CHIKV/ZIKV | 1 | neg | neg |
| | 7dpi | CHIKV/DENV | 42 | C, D | C |
| | | CHIKV/DENV | 52 | C, D | C, D |
| | | CHIKV/DENV | 54 | C, D | C, D |
| | | CHIKV/DENV | 41 | neg | neg |
| | | CHIKV/ZIKV | 53 | C, Z | C, Z |
| | | CHIKV/ZIKV | 54 | C, Z | C, Z |
| | | CHIKV/ZIKV | 44 | C | C |
| | | CHIKV/ZIKV | 45 | C | C |
| | | CHIKV/ZIKV | 41 | neg | neg |
| | 14dpi | CHIKV only | 91 | C | C |
| | | CHIKV only | 92 | C | none |
| | | CHIKV only | 93 | C | none |
| | | DENV only | 90 | D | D |
| | | DENV only | 99 | D | D |

| | | | | | |
|---|---|---|---|---|---|
| | | DENV only | 101 | D | none |
| | | ZIKV only | 81 | Z | Z |
| | | ZIKV only | 88 | Z | Z |
| | | ZIKV only | 89 | Z | Z |
| | | CHIKV/DENV | 82 | C, D | D |
| | | CHIKV/DENV | 83 | C, D | C, D |
| | | CHIKV/DENV | 90 | C, D | C, D |
| | | CHIKV/DENV | 93 | C, D | C, D |
| | | CHIKV/DENV | 98 | C, D | D |
| | | CHIKV/DENV | 105 | C, D | D |
| | | CHIKV/DENV | 116 | C, D | D |
| | | CHIKV/DENV | 120 | C, D | neg |
| | | CHIKV/DENV | 81 | neg | neg |
| | | CHIKV/ZIKV | 88 | C, Z | Z |
| | | CHIKV/ZIKV | 100 | C, Z | neg |
| | | CHIKV/ZIKV | 87 | C | C |
| | | CHIKV/ZIKV | 95 | C | neg |
| | | CHIKV/ZIKV | 85 | Z | Z |
| | | CHIKV/ZIKV | 107 | Z | neg |
| | | CHIKV/ZIKV | 81 | neg | neg |
| | | DENV/ZIKV | 81 | D, Z | neg |
| | | DENV/ZIKV | 82 | D, Z | D, Z |
| | | DENV/ZIKV | 85 | D, Z | Z |
| | | DENV/ZIKV | 93 | D, Z | D |
| | | DENV/ZIKV | 108 | D, Z | D, Z |
| | | DENV/ZIKV | 116 | D, Z | D, Z |
| | | DENV/ZIKV | 119 | D, Z | D, Z |
| | | DENV/ZIKV | 90 | D (Z ct>36.5) | D, Z |
| | | DENV/ZIKV | 106 | D | D |
| | | DENV/ZIKV | 112 | D | D |
| | | DENV/ZIKV | 120 | D | D |
| | | DENV/ZIKV | 83 | neg | neg |
| **Triple infection** | 14dpi | Triple | 2 | C, D, Z | C, D, Z |
| | | Triple | 5 | C, D, Z | C, D, Z |
| | | Triple | 14 | C, D, Z | C, D, Z |
| | | Triple | 15 | C, D, Z | C, Z |
| | | Triple | 16 | C, D, Z | D, Z |
| | | Triple | 17 | C, D, Z | C, D, Z |
| | | Triple | 32 | C, D | none |
| | | Triple | 45 | C, D | C |
| | | Triple | 48 | D, Z | D, Z |
| | | Triple | 3 | neg | neg |
| | 21dpi | Triple | 4 | D, Z | Z |
| | | Triple | 22 | D, Z | D, Z |
| | | Triple | 36 | D, Z | D, Z |
| | | Triple | 43 | C, Z | Z |
| | | Triple | 29 | C, D, Z | Z |
| | | Triple | 48 | C, D, Z | D, Z |
| | | Triple | 14 | Neg | neg |
| | | Triple | 15 | neg | neg |

* virus abbreviations: C =CHIKV; D=DENV-2; Z=ZIKV

**Supplementary Table 2.** Primers and probes used for multiplex qRT-PCR.

| Primer name | Sequence |
|---|---|
| **Zika1087_CDC_F[1]** | CCGCTGCCCAACACAAG |
| **Zika1108_CDC_FAM[1]** | AGCCTACCTTGACAAGCAGTCAGACACTCAA |
| **Zika1163_CDC_R[1]** | CCACTAACGTTCTTTTGCAGACAT |
| **CHIKV_qRT-PCR_JF_F[2]** | CACCCGAAGTAGCCCTGAATG |
| **CHIKV3288_Cy5[2]** | GAGAATAGCCCGCTGTCTAGATCCAC |
| **CHIKV_qRT-PCR_JF_R[2]** | TCCGAACATCTTTCCTCCCG |
| **DENV2-Merida_4198F** | TCGTATGGAGGAGGCTGGAA |
| **DENV2-Merida_4305R** | GGGTTTTGTTTGGACGGCTC |
| **DENV2-Merida_4219_HEX** | CTAGAAGGAGAATGGAAGGAAGGAG |
| **CHIKV_T7_2388F** | TAATACGACTCACTATAGGGCGCTGCTCTTGAATGGATGC |
| **CHIKV_T7_3505R** | TAGTCTCCTGTTGGCCGGTA |
| **DENV2-Merida_T7_3962F** | TAATACGACTCACTATAGGGAACGGGCTGGAGTATTGTGG |
| **DENV2-Merida_T7_4840R** | CTGGGTCTGTGAAATGGGCT |

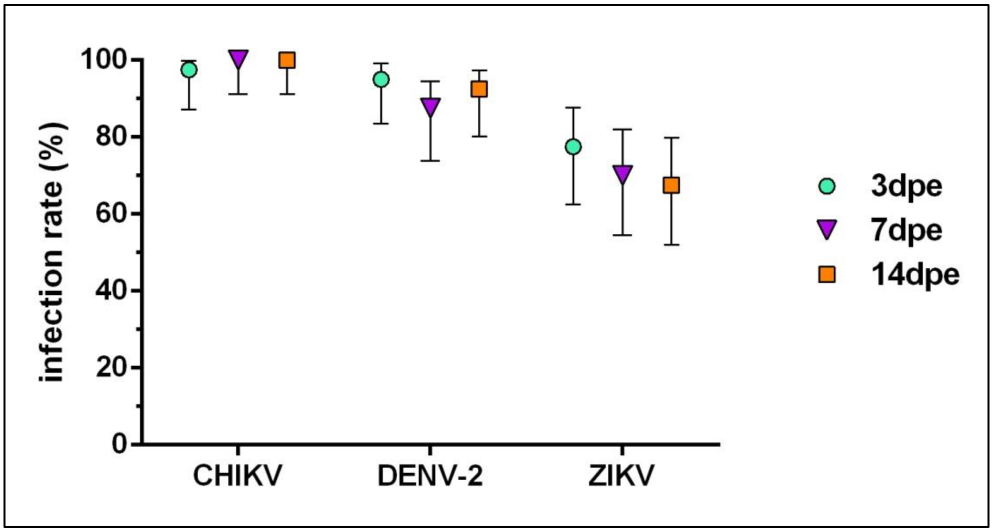


**Supplementary Figure 1. Infection rates are comparable across time points**. Infection rates for CHIKV, DENV-2 and ZIKV at all time points during single infection from one representative experiment (Experiment 4, see Table 3). Each data point indicates the percentage of infected mosquitoes (n=40 per condition/time point) and error bars indicate 95% CI. No significant differences were found between time points ($p>0.05$; Fisher's exact test).

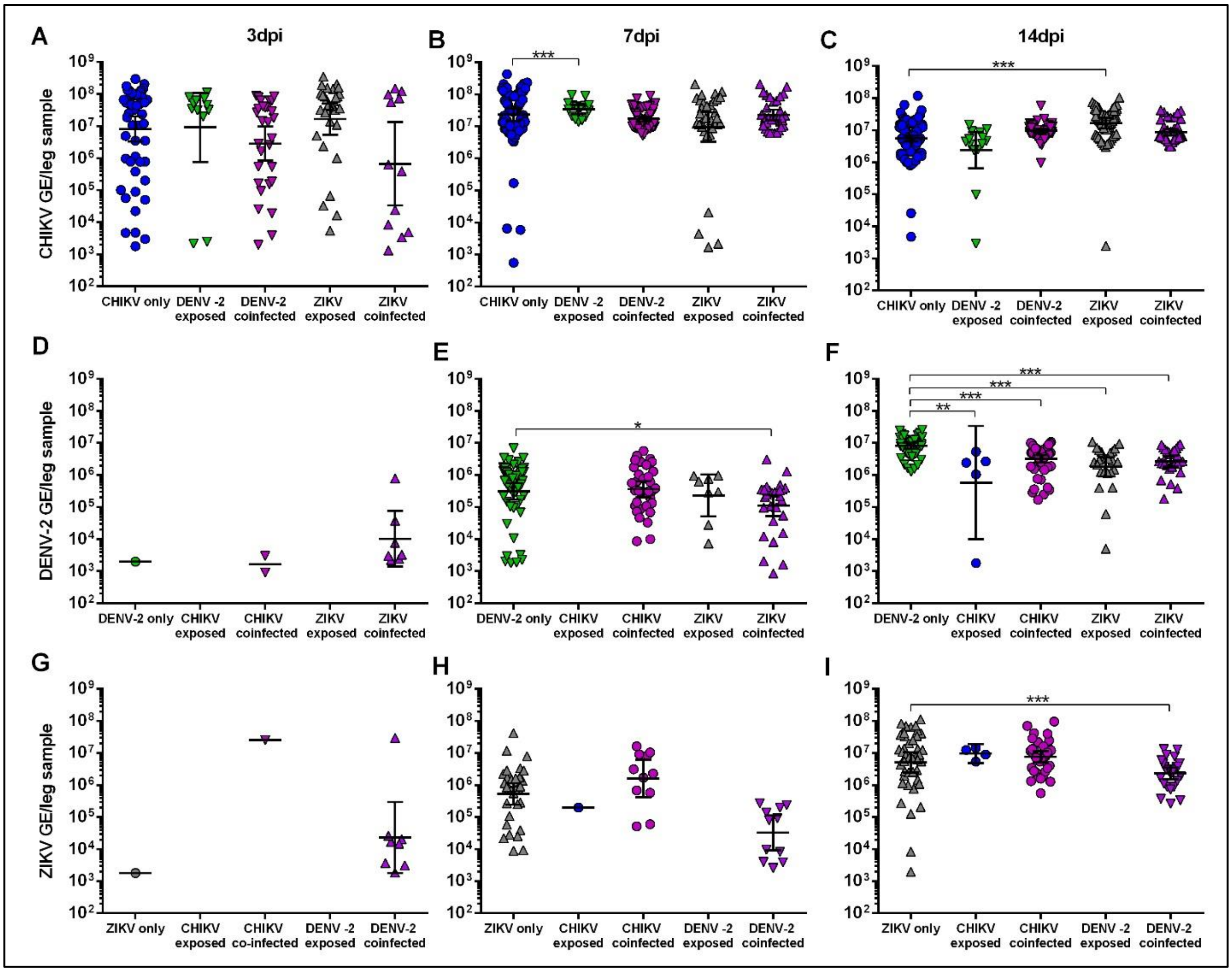


**Supplementary Figure 2. RNA levels in legs positive for CHIKV, DENV-2 and/or ZIKV.** GE/legs are shown for samples positive for CHIKV (A, B, C), DENV-2 (D, E, F) and ZIKV (G, H, I) at 3dpi (A, D, G), 7dpi (B, E, H) and 14dpi (C, F, I). 'Exposed' indicates samples that were co-exposed with the named virus without resulting in coinfection. 'Coinfected' samples were positive for both viruses. The geometric mean is indicated and error bars represent the 95% confidence interval. Statistical significance was determined using one-way ANOVA with multiple comparisons (*p<0.05; **p<0.01; ***p<0.0001).

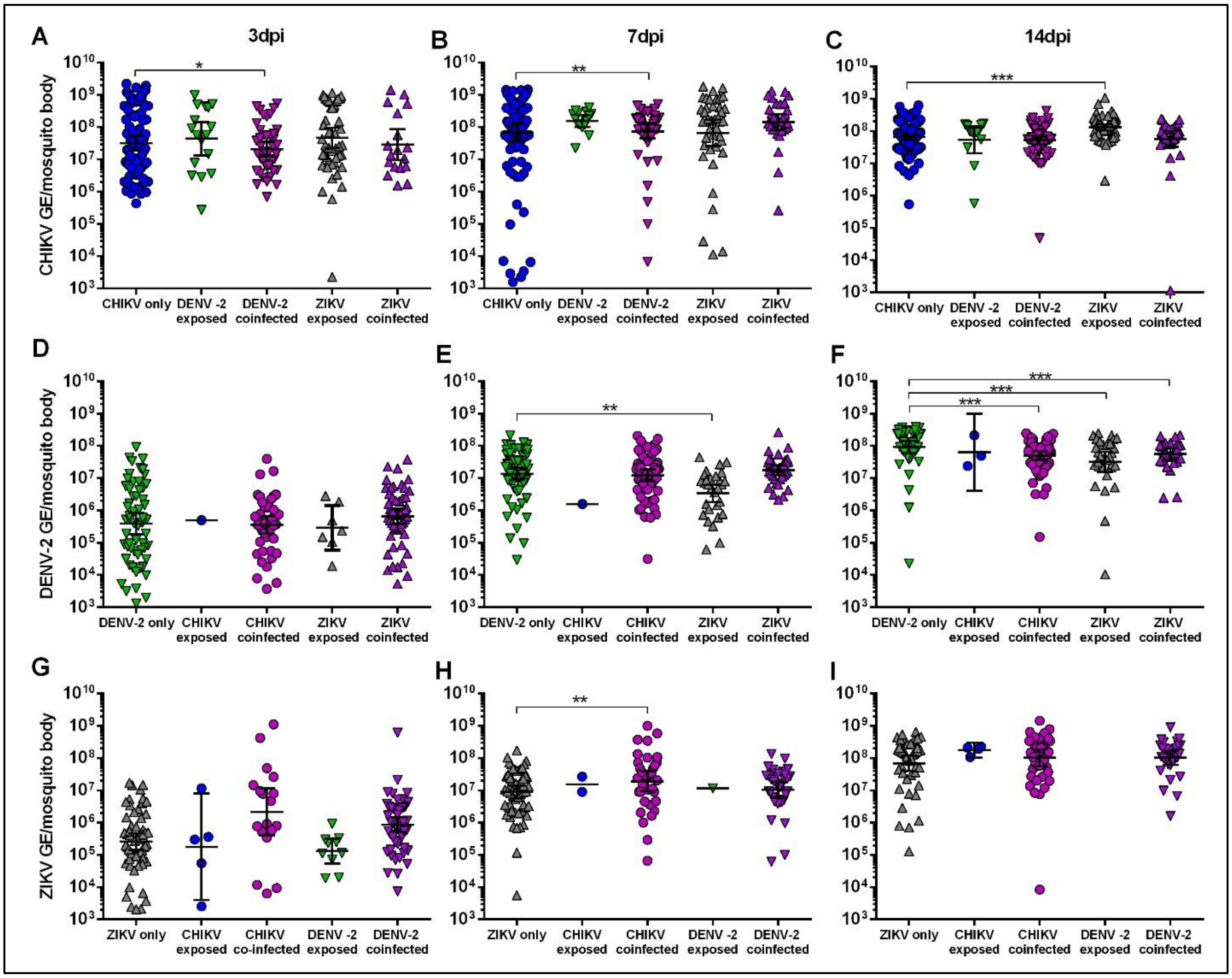


**Supplementary Figure 3. RNA levels in bodies positive for CHIKV, DENV-2 and/or ZIKV.** GE/body are shown for samples positive for CHIKV (A, B, C), DENV-2 (D, E, F) and ZIKV (G, H, I) at 3dpi (A, D, G), 7dpi (B, E, H) and 14dpi (C, F, I). 'Exposed' indicates samples that were co-exposed with the named virus without resulting in coinfection. 'Coinfected' samples were positive for both viruses. The geometric mean is indicated and error bars represent the 95% confidence interval. Statistical significance was determined using one-way ANOVA with multiple comparisons (*p<0.05; **p<0.01; ***p<0.0001).

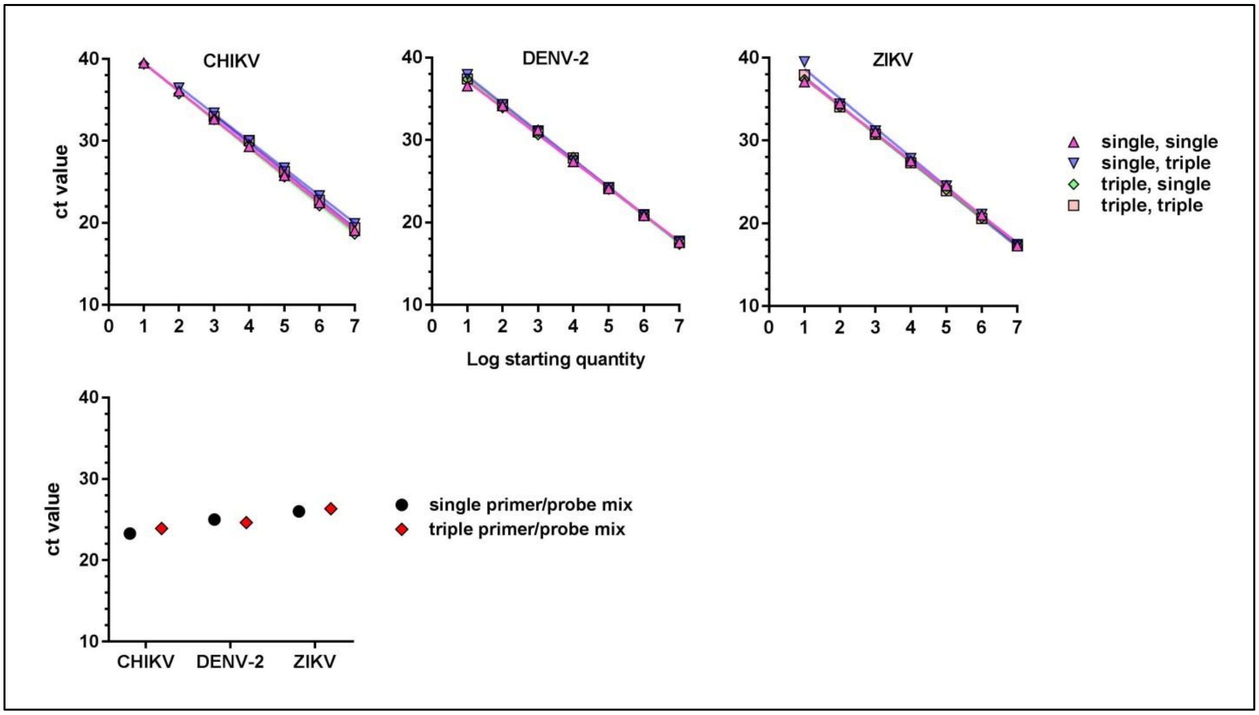


**Supplementary Figure 4. Multiplex qRT-PCR sensitivity and specificity.** qRT-PCR was performed as described (see methods) to quantify in parallel CHIKV (A), DENV-2 (B) and ZIKV (C) RNA standards. The four different conditions were as follows: single virus standards with single primer/probe mix (single, single); single virus standards with triple primer/probe mix (single, triple); triple virus standards with single primer/probe mix (triple, single); triple standards with triple primer/probe mix (triple, triple). Panel D shows representative quantification using the three single primer/probe mixes as well as a multiplexed triple primer/probe mix from a triple positive leg sample . Single and triple primer/probe mix results for each virus were within a 0.65 ct value difference.

## Supplementary References

1 Lanciotti, R. S. *et al.* Genetic and serologic properties of Zika virus associated with an epidemic, Yap State, Micronesia, 2007. *Emerging infectious diseases* **14**, 1232-1239, doi:10.3201/eid1408.080287 (2008).

2 Grubaugh, N. D. *et al.* Xenosurveillance: a novel mosquito-based approach for examining the human-pathogen landscape. *PLoS neglected tropical diseases* **9**, e0003628, doi:10.1371/journal.pntd.0003628 (2015).
